# Refining the Multidimensional Measure of Coping for Adolescents: Psychometric Validation of a Short Form and Its Higher-Order Structure in Chinese Adolescents

**DOI:** 10.3390/bs16030392

**Published:** 2026-03-09

**Authors:** Bin Yuan, Shasha Qiu, Caina Li

**Affiliations:** 1 School of Psychology, Shaanxi Normal University, Shaanxi Provincial Key Research Center of Child Mental and Behavioral Health, Xi’an 710062, China; yuanbin329@snnu.edu.cn (B.Y.); qiushasha@hpu.edu.cn (S.Q.); 2 Mental Health Education and Counseling Center, Henan Polytechnic University, Jiaozuo 454003, China

**Keywords:** Multidimensional Measure of Coping, higher-order structure, short-form scale, psychometric validation, Chinese adolescents

## Abstract

How students cope with academic stress is crucial for learning and well-being. The Multidimensional Measure of Coping (MMC) provides a comprehensive hierarchical assessment of academic coping; however, its length and adaptive–maladaptive distinction may limit practical use and constrain a more differentiated understanding of academic coping. This study aimed to refine the MMC and propose a differentiated higher-order structure for the MMC-Short Form (MMC-SF). Data were drawn from three adolescent samples from Northwest China (2024–2025): an exploratory sample (N = 1342), a confirmatory sample (N = 2037; test–retest N = 367; 4 weeks), and a longitudinal sample (T1 N = 948; T2 N = 760 at 1 month; T3 N = 893 at 6 months). Psychometric analyses (item analysis, exploratory structural equation modeling and confirmatory factor analysis [CFA]) demonstrated that the 34-item MMC-SF reliably preserved the original 11-factor structure. Furthermore, a multi-method investigation integrating higher-order exploratory factor analysis and second-order CFA supported a hybrid higher-order structure, with proactive engagement and defensive disengagement as higher-order dimensions and escape coping as a distinct first-order factor. The predictive validity was examined in relation to academic self-efficacy and burnout. These findings support the reconceptualization of academic coping and provide a brief, psychometrically robust assessment tool.

## 1. Introduction

Academic life is a pivotal developmental context in adolescence (Eccles & Roeser, 2011). From a transactional perspective, students’ adjustment reflects not only exposure to academic stress but also coping, which is defined as the cognitive and behavioral efforts to manage demands appraised as exceeding one’s resources (Compas et al., 2001; Lazarus & Folkman, 1984). In school settings, coping functions as a central mechanism linking academic stressors to achievement, school functioning, and psychological well-being (Modecki et al., 2017; Putwain et al., 2024; E. A. Skinner & Saxton, 2019).

The Multidimensional Measure of Coping (MMC; E. Skinner et al., 2013) was developed to capture this domain-specific process within a hierarchical structure encompassing concrete coping ways, 11 functionally organized first-order families (e.g., strategizing, help-seeking, rumination, and escape), and higher-order composites traditionally labeled adaptive–maladaptive. Unlike domain-general scales adapted with academic instructions (e.g., the Coping Orientation to Problems Experienced [COPE] scale; Carver et al., 1989, as used by Parada & Verlhiac, 2021), the MMC anchors responses in school-specific stressor scenarios (e.g., “difficulties on an important test”), eliciting contextually grounded actions (e.g., “I get help to understand the material better”). This design addresses coping domain sensitivity (Aldwin, 2007; Somerfield & McCrae, 2000) and strengthens construct representation and the validity argument for interpreting scores in academic contexts (American Educational Research Association et al., 2014; Messick, 1995).

This hierarchical structure bridges a persistent tension in coping assessment: broad functional categories can obscure situated actions, whereas behavior-level lists remain theoretically unmoored (Patterson & McCubbin, 1987; E. A. Skinner et al., 2003; Tobin et al., 1989). The MMC has been rigorously validated in multiple linguistic versions (e.g., Gonçalves et al., 2019; Morales-Castillo, 2022) and widely used from late childhood through adolescence to map teachable coping actions onto functionally coherent families, thereby linking behavioral specificity with higher-order organization (Raine et al., 2023; Tu et al., 2020; Zhang & Huang, 2025). However, this scale encounters several challenges. At the first-order level, the classification into 11 coping families, while comprehensive, may result in conceptual fragmentation and a weakly integrated structure. Moreover, the 55 items may impose a response burden in practical application (Edwards et al., 2004; Rolstad et al., 2011), particularly due to redundancy within coping families (e.g., multiple rumination items measuring persistent thinking, such as “I can’t stop thinking about it” and “I keep thinking about it over and over”). This redundancy may explain why some studies use only narrow subsets of coping families but still report overall adaptive and maladaptive composites (Li et al., 2022), which limits construct coverage and comparability across studies.

At the higher-order level, the adaptive–maladaptive dichotomy, centered on academic re-engagement (E. Skinner et al., 2013), may not fully capture the contextual nature of coping effectiveness (Compas et al., 2017; Frydenberg, 1997; Lazarus & Folkman, 1984). Coping flexibility research supports that effectiveness depends on strategic fit and adjustment over time, not on the use of universally “good” strategies (Bonanno & Burton, 2013; C. Cheng et al., 2014; Leslie-Miller et al., 2025; Waugh et al., 2020). These issues have led to recent efforts to refine both measurement and higher-order organization, such as retaining two items per coping family, resulting in a proposed second-order hybrid model that comprises two higher-order dimensions: engagement and disengagement, and two distinct first-order factors: comfort-seeking and escape (Zimmer-Gembeck et al., 2023). Taken together, these considerations call for moving beyond fixed evaluative categories toward a context-dependent understanding of coping.

In academic environments, stress tends to be cumulative due to persistent evaluation and social comparison (E. Skinner et al., 2013), particularly within the high-pressure Chinese educational context (Y. Cheng & Hamid, 2025). In this sense, daily academic demands operate as immediate pressures, whereas the attainments students value most—such as academic standing, social recognition, and a stable sense of self-efficacy—typically emerge and solidify gradually over time. A key challenge for academic coping is how adolescents manage their psychological resources at the intersection of these immediate pressures and protracted outcomes. This discrepancy in timescales creates a fundamental tension in students’ coping efforts. Consequently, strategies traditionally seen as maladaptive can serve functional roles in context: for instance, strategic avoidance may act as a tactical pause to preserve energy (Wadsworth, 2015), and rumination may facilitate meaning-making rather than merely reflecting distress (Watkins, 2008). This complex relationship between coping behaviors and their context-dependent functions raises a pivotal question: How might we construct a framework for academic coping that more accurately represents the functional purposes of student coping under chronic stress?

The Conservation of Resources (COR) theory (Hobfoll, 1989) offers a foundational lens for understanding coping as a functional, process-oriented form of resource manage-ment. Beyond a stress management model, it establishes an analytic paradigm centered on resources. COR proposes that stress emerges when valued resources are threatened or lost, or when resource investment fails to yield expected gains. Coping, in turn, refers to efforts to conserve, protect, and acquire resources. Accordingly, the key to evaluating coping strategies lies not in process-based labels (e.g., problem-focused vs. emotion-focused coping) or static outcome classifications (e.g., adaptive vs. maladaptive), but in the resource-management goals they serve. Drawing on the COR theory, individuals’ coping strategies can be summarized as serving three primary goals, depending on contextual factors such as resource availability and threat controllability: resource acquisition, resource protection, and resource reallocation (Hobfoll, 1989; Holmgreen et al., 2017).

This perspective effectively explains coping with academic stress: such stress is persistent and continually depletes students’ finite resources like time and mental energy, compelling them to proactively acquire, cautiously conserve, or flexibly reallocate these valuable assets. Consequently, this theoretical framework provides a fitting and robust lens for understanding adolescents’ complex coping behaviors as they navigate daily academic demands and evaluative pressures.

In light of these challenges, the goal of this study was to revise and validate a functionally oriented and culturally adaptive framework for academic coping, namely, the MMC-Short Form (MMC-SF). We retained the original 11 coping families with fewer items and proposed a reconfigured higher-order model to enhance integration and coherence. Using three independent samples of Chinese adolescents, we employed exploratory factor analysis (EFA) and confirmatory factor analysis (CFA) to examine the factor structure and compare models. The predictive utility of the resulting structure, the MMC-SF, for academic self-efficacy and burnout was assessed at multiple time points.

## 2. Materials and Methods

### 2.1. Participants and Procedure

This study used three independent samples of adolescents from Northwest China collected during the 2024–2025 academic year. The participants were recruited from multiple schools. Within each school, students completed a survey during regular class sessions under the supervision of trained administrators who provided standardized instructions and monitored completion.

Sample 1: Exploratory Sample

This sample was used for exploratory analyses for the revision of the MMC and included both junior and senior high school students. The total sample comprised 1342 participants (49.4% male; overall *M*_age_ = 14.48, standard deviation [*SD*] = 2.84). Among them, 650 were junior high school students (46.3% male; *M*_age_ = 12.94, *SD* = 0.74) and 692 were senior high school students (52.3% male; *M*_age_ = 15.81, *SD* = 0.77).

Sample 2: Confirmatory Sample

This sample was designated for confirmatory analyses for the revision of the MMC and included both junior and senior high school students. The total sample comprised 2037 participants (50.2% male; overall *M*_age_ = 14.36, *SD* = 1.55). Of these, 935 were junior high school students (49.0% male; *M*_age_ = 12.80, *SD* = 0.48) and 1102 were senior high school students (51.2% male; *M*_age_ = 15.68, *SD* = 0.68). A subsample (N = 367; 4-week follow-up) completed the MMC-SF again four weeks later to assess test–retest reliability.

Sample 3: Longitudinal Sample

A longitudinal sample consisting of first-year senior high school students was used to examine predictive validity. Valid responses were obtained from 948 participants at baseline (T1: 49.8% male, *M*_age_ = 15.38, *SD* = 0.55), 760 participants one month later (T2: 49.6% male, *M*_age_ = 15.43, *SD* = 0.56), and 893 participants six months later (T3: 47.5% male, *M*_age_ = 15.90, *SD* = 0.55). Attrition across waves was primarily due to student absences or scheduling conflicts; intermittent missingness led to some T2 non-responders returning at T3. Little’s Missing Completely At Random (MCAR) test indicated that the missing data pattern conformed to a completely random mechanism, χ^2^(648) = 677.58, *p* = 0.204.

All procedures were approved by the university’s ethics committee. Informed consent was obtained from school administrators, parents, and all participating students. Permission to revise the MMC was obtained from the original authors.

### 2.2. Measures

Academic Coping: Academic coping was assessed using the MMC (E. Skinner et al., 2013). In this study, students responded to items with reference to four typical academic situations, such as “when I have problems in an important exam” and “when I have difficulties in a particular subject.” The original MMC includes 55 items covering 11 first-order families (five adaptive and six maladaptive) rated on a 4-point Likert scale (1 = Not at all true for me; 4 = Very true for me), with higher scores indicating more frequent use of the strategy. Across the present sample, the subscale reliabilities ranged from 0.87 to 0.93. In line with the original framework, these coping families represent theoretically coherent groupings of items; in the present study, we refer to them as first-order factors when describing our statistical models.

Academic Self-Efficacy: Academic self-efficacy was measured using the self-efficacy subscale of the Self-Regulated Learning Strategy Questionnaire developed by Kong and Lu (2012), which was adapted from the Motivated Strategies for Learning Questionnaire (MSLQ; Pintrich et al., 1993). The subscale consists of seven items rated on a 7-point Likert scale (1 = not at all true of me, 7 = very true of me), with higher scores indicating higher levels of academic self-efficacy. In this study, Cronbach’s α ranged from 0.958 to 0.962 across samples.

Academic Burnout: Academic burnout was measured using the School Burnout Inventory (SBI; Salmela-Aro et al., 2009), which assesses students’ feelings of strain and exhaustion related to their schoolwork. The scale consists of 9 items forming three dimensions: exhaustion, cynicism, and feelings of inadequacy (lack of efficacy). Items are rated on a 6-point Likert scale (1 = completely disagree, 6 = strongly agree), with higher scores indicating higher levels of academic burnout. In this study, Cronbach’s α ranged from 0.958 to 0.960 across samples.

### 2.3. Data Analyses

All analyses were conducted using Mplus 8.3 and R 4.3.1 (two-tailed, α = 0.05). In Mplus, models were estimated using maximum likelihood estimation (MLR), full information maximum likelihood (FIML), and exploratory structural equation modeling (ESEM) with target rotation.

Stage 1: Revision and Validation of the First-Order Structure

In Stage 1, the proposed MMC-SF was refined and validated while retaining 11 first-order factors. Item reduction followed the short-form development guidelines (Smith et al., 2000) and SEM-based item screening criteria (Byrne, 2012; Hair et al., 2009). In Sample 1, we evaluated the psychometric quality of the original 55-item MMC. Given that coping strategies in real settings often overlap and co-occur functionally, we employed ESEM (MLR, target rotation; Asparouhov & Muthén, 2009) to accommodate cross-loadings, thereby capturing a more realistic and conceptually nuanced representation of the coping construct during item screening. This approach allowed us to identify and remove items with weak measurement properties or conceptual overlap based on empirical evidence and theoretical considerations. The resulting item set was then tested in Sample 2 using 11-factor CFA (MLR) to confirm the first-order structure and evaluate the psychometric adequacy of the MMC-SF in an independent sample.

Stage 2: Identification and Validation of the Higher-Order Functional Structure

To examine the broader organization of academic coping, we first estimated the inter-factor correlation matrix (Phi) among the 11 coping factors in Sample 1 for later analyses. The Phi matrix reflects the correlation structure among the first-order factors and provides a construct-level basis for examining higher-order organization (Wolff & Preising, 2005) while reducing the influence of item-specific variance, consistent with transactional and developmental perspectives that view coping as an interconnected set of responses rather than isolated strategies.

Our theoretical framework suggests that coping may be organized into a limited number of higher-order functional dimensions. To determine the number of higher-order factors, we first examined the eigenvalue pattern (Kaiser, 1960) and then conducted parallel analysis (Horn, 1965; Hayton et al., 2004) in Sample 1. Higher-order EFA was then conducted using maximum likelihood extraction with direct oblimin rotation to explore the latent higher-order structure (Fabrigar et al., 1999). To validate this higher-order structure, second-order CFA (Marsh et al., 2014) was tested in Sample 2 to ensure that the first-order factors were appropriately grouped into higher-order dimensions. The model comparisons considered fit indices (comparative fit index [CFI], Tucker–Lewis index [TLI], root mean square error of approximation [RMSEA]), and theoretical interpretability.

In Sample 3, longitudinal regressions tested whether the three higher-order coping dimensions were prospectively associated with academic self-efficacy and burnout over one-month (T1–T2) and six-month (T1–T3) intervals, controlling for T1 self-efficacy and burnout. Missing data were addressed via FIML in Mplus.

## 3. Results

### 3.1. The First-Order Structure: Revision and Validation of the MMC-SF

#### 3.1.1. Initial Evaluation and Item Reduction

**Figure 1:**
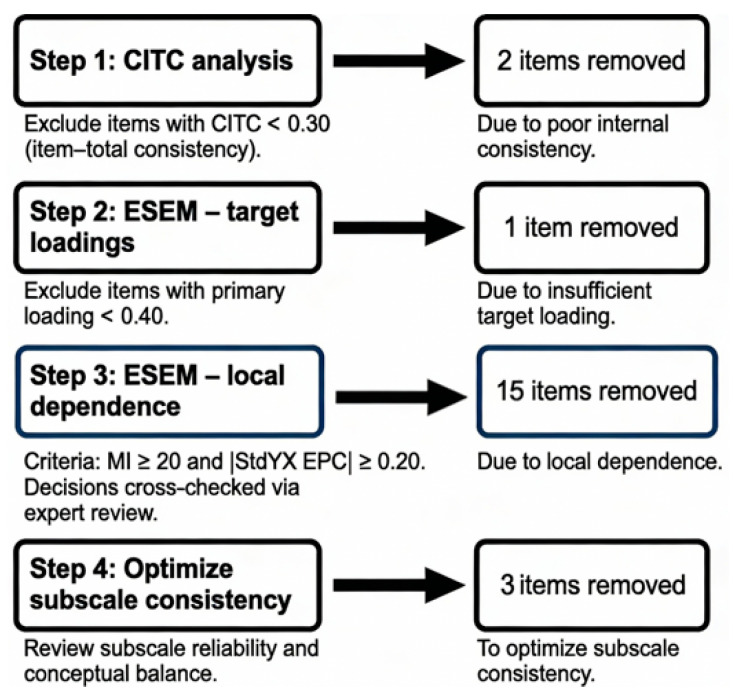
MMC-SF Item Reduction Workflow. Note: CITC = Corrected Item-Total Correlation; ESEM = exploratory structural equation modeling.

The psychometric evaluation of the original 55-item MMC in Sample 1 indicated good to excellent internal consistency across subscales (α = 0.87–0.93) and acceptable to adequate fit for the baseline 11-factor model (RMSEA = 0.055, CFI = 0.904, TLI = 0.896), motivating item refinement. Item reduction followed the workflow summarized in Figure 1. The reduction process comprised four sequential steps: (1) CITC analysis (2 items removed, CITC < 0.30); (2) ESEM target loading screening (1 item removed, loading < 0.40); (3) ESEM local dependence testing (15 items removed, MI ≥ 20, |EPC| ≥ 0.20); (4) subscale optimization (3 items removed, for reliability and conceptual balance). Item removal decisions were reached through discussion between the first and second authors, with any disagreements resolved by consulting a professor of psychology for final adjudication. The resulting MMC-SF retained the original 11-factor structure, with 3–4 items per factor (34 items in total), representing an approximately 38% reduction in length.

#### 3.1.2. Structural Validation of the First-Order Model

In the validation sample (Sample 2; N = 2037), the results in Table 1 indicate that the MMC-SF fit the data substantially better than the full MMC under both ESEM and CFA. Under ESEM, the MMC-SF showed a near-perfect global fit (CFI = 0.998, RMSEA = 0.029), outperforming the full MMC (CFI = 0.959, RMSEA = 0.039). The same advantage was observed in the CFA, where the MMC-SF achieved a clearly stronger fit (CFI = 0.980, RMSEA = 0.028) than the full MMC (CFI = 0.928, RMSEA = 0.042), supporting the improved structural representation of the MMC-SF.

The item-level factor loadings for the final MMC-SF were evaluated using an 11-factor ESEM (Appendix A, Table A1). Primary loadings were generally high (λs ranged from 0.47 to 0.91), with most exceeding 0.50. The cross-loadings were generally small; when present, the primary loadings exceeded the largest cross-loading by at least 0.20. Given that ESEM permits cross-loadings, this pattern suggests a limited, theoretically plausible overlap among closely related factors and supports a well-differentiated 11-factor structure.

Descriptive statistics and intercorrelations for the 11 first-order MMC-SF subscales in Sample 2 (N = 2037) are presented in Table 2. Subscale means ranged from 1.78 to 3.17 (*SD*s = 0.68–0.93), and skewness and kurtosis were generally within ±1, indicating no severe departures from normality and adequate score variability. The correlations suggested two coherent groupings: strategizing, help-seeking, comfort-seeking, self-encouragement, and commitment tended to be moderately to strongly interrelated, whereas confusion, concealment, self-pity, rumination, and projection showed a separate pattern of moderate to strong intercorrelations. Escape displayed a more diffuse pattern, with small to moderate correlations with both groupings.

Evidence for reliability was also strong: all subscales displayed high internal consistency and generally moderate four-week test–retest stability in a subsample (N = 367). Consistent with its more diffuse correlational profile as noted, escape emerged as the most distinctive dimension and exhibited the weakest stability (intraclass correlation coefficient [ICC] = 0.25). Together, these findings indicated improved structural fit and dependable subscale scores, justifying further evaluation of the validity and measurement invariance of the MMC-SF.

#### 3.1.3. Measurement Invariance

The 11-factor MMC-SF demonstrated scalar invariance across gender and school level (junior vs. senior high school), supporting latent mean comparisons (Table 3). Residual (strict) invariance was acceptable for gender based on ΔCFI/ΔTLI, although the SRMR increased at the residual step. In contrast, strict invariance was not supported across school levels, where fit deteriorated markedly at the residual-constrained step (ΔCFI = −0.041; ΔSRMR = 0.136), suggesting differences in item residual variances between junior and senior high school students.

### 3.2. The Higher-Order Structure: Identification and Validation of the Functional Model

#### 3.2.1. Exploration of the Higher-Order Structure

In the exploratory sample (Sample 1), the inter-factor correlation matrix was suitable for higher-order factor analysis (Kaiser-Meyer-Olkin [KMO] = 0.827; Bartlett’s χ^2^(55) = 5722.68, *p* < 0.001). The eigenvalue pattern suggested a limited number of broad dimensions, with two eigenvalues exceeding 1 (λ_1_ = 3.24, 29.46%; λ_2_ = 3.13, 28.41%) and a third approaching unity (λ_3_ = 0.94, 8.50%). Parallel analysis based on the 95th percentile criterion supported retaining four higher-order factors (λ_4_ = 0.13, p95 = 0.08; λ_5_ = 0.03, p95 = 0.05). Considering the original scale’s two-factor structure and the current indication of three to four potential factors, we systematically tested two-, three-, and four-factor models to identify the optimal structure.

Table 4 reports standardized pattern coefficients from higher-order EFAs across alternative solutions. The two-factor solution showed an interpretive inconsistency, as escape loaded weakly (λ = 0.36) and aligned with the factor dominated by dimensions that were originally classified as adaptive. The three-factor solution clarified this pattern, with escape forming a distinct third factor (λ = 0.49). In the four-factor solution, help-seeking (λ ≈ 0.98) was largely isolated without improving overall coherence, while projection’s cross-loadings persisted (primary loading 0.48 vs. 0.38). Although projection cross-loaded, its strongest loading was on disengagement rather than escape, supporting its retention there.

Although parallel analysis (95th percentile) suggested four factors, the four-factor solution showed signs of overfactoring and offered no conceptual advantage over the three-factor solution; therefore, we retained the more interpretable and parsimonious three-factor hybrid structure for subsequent analyses: (1) proactive engagement (strategizing, help-seeking, comfort-seeking, self-encouragement, and commitment), (2) defensive disengagement (confusion, concealment, self-pity, rumination, and projection), and (3) escape coping, retained as a distinct first-order factor.

#### 3.2.2. Confirmatory Validation of the Higher-Order Model

The CFA indicated that the three-factor hybrid model provided an excellent fit (CFI = 0.955, RMSEA = 0.044, SRMR = 0.053) and improved the two-factor alternative. Although its fit was comparable to that of the four-factor model, the three-factor solution was more parsimonious and slightly favored by the information criteria, and offered clearer theoretical interpretability (Table 5). Accordingly, this structure was adopted as the final model.

The reliability estimates indicated strong internal consistency for the higher-order structure. The two higher-order factors showed excellent internal consistency (engagement: ω_total = 0.95, α = 0.95, ICC = 0.53; disengagement: ω_total = 0.96, α = 0.96, ICC = 0.64). The escape factor showed strong internal consistency (ω_total = 0.82, α = 0.81) but lower temporal stability (ICC = 0.25).

#### 3.2.3. Criterion Validity and Predictive Utility

Table 6 summarizes baseline-adjusted hierarchical regressions linking the three higher-order coping dimensions to academic self-efficacy and burnout over one-month and six-month intervals.

Proactive engagement was prospectively associated with higher self-efficacy at one and six-months (β = 0.192, 0.155) and showed marginal negative associations with burnout (β = −0.078, −0.091; *p* = 0.072, 0.052), whereas defensive disengagement showed the opposite pattern, with lower self-efficacy (β = −0.182, −0.165) and higher burnout (β = 0.230, 0.201) across both intervals. Notably, escape coping showed a small, time-limited positive association with self-efficacy at one-month (β = 0.120, *p* = 0.01), an effect that had dissipated by six-months (β = 0.026, *p* = 0.595) and that did not extend to burnout.

These differential patterns validate the conceptual distinction among the three higher-order coping dimensions and highlight their unique associations with academic adjustment. Taken together, these findings support the MMC-SF as a feasible and psychometrically sound tool, and the higher-order framework offers a more nuanced account of Chinese adolescents’ academic coping than a simple adaptive–maladaptive dichotomy.

## 4. Discussion

### 4.1. Refining the Structure of the MMC-SF in Relation to Academic Coping

From a developmental perspective, coping patterns that adolescents develop in response to school stress may map onto broader developmental tasks, such as emotion regulation, self-system development, relationships, and meaning in life (Compas et al., 2017; E. A. Skinner & Saxton, 2019; Ward et al., 2023). Accordingly, a functional perspective is essential, as it connects coping to outcomes both within and beyond school, thereby reflecting the cross-domain nature of adolescent adaptation (E. A. Skinner & Zimmer-Gembeck, 2023).

At the first-order level, the MMC-SF (34-item) substantially reduced the respondent burden while maintaining high internal consistency and clear differentiation among first-order factors (coping families), demonstrating its suitability for large-scale or longitudinal research. Measurement invariance tests showed strict invariance across gender but only achieved scalar invariance across school stages. This suggests that although the core coping construct remains consistent, its functional expression may evolve with development (Meredith, 1993; Millsap & Yun-Tein, 2004). In early adolescence, coping is more closely tied to immediate peer and situational demands; whereas in later adolescence it becomes more stable and self-directed, aligned with personal goals (E. A. Skinner & Zimmer-Gembeck, 2007). Age-related advances in cognitive control (Luna et al., 2015), emotion regulation (Zimmermann & Iwanski, 2014) and resistance to peer pressure (Steinberg & Monahan, 2007) may help explain this developmental progression in coping patterns. These further underscores the necessity of adopting a dynamic, developmental, and functional perspective on coping, as its expression evolves across adolescence.

At the higher-order level, the first-order factors coalesced into a three-domain hybrid structure, namely, proactive engagement, defensive disengagement, and escape coping. These domains represent functionally distinct response modes, each serving to regulate personal and contextual resources—whether by investing effort toward goal pursuit (proactive engagement), engaging in resource-conserving but often self-entangling internal regulation (defensive disengagement), or redirecting attention and resources to alternative goals (escape coping). These domains capture distinct resource-regulatory modes, aligning more close with the situated, resource-sensitive reality of students’ coping than the original adaptive–maladaptive dichotomy.

### 4.2. Proactive Engagement: Resource Investment and Approach-Oriented Coping

Across school-based coping research, a consistent pattern is that problem solving and constructive support seeking tend to relate to stronger academic functioning, including higher self-efficacy and greater engagement and buoyancy (e.g., E. A. Skinner & Saxton, 2019; Modecki et al., 2017; Putwain et al., 2024). In line with this broader evidence, proactive engagement in the MMC-SF, indexed by strategizing, help-seeking, comfort-seeking, self-encouragement, and commitment, showed positive associations with academic self-efficacy at both the one-month and six-month follow-ups in our longitudinal analyses.

From the standpoint of COR, these strategies can be understood as intentional resource investments that build and protect resource caravans within supportive learning environments (Hobfoll, 2011), with positive emotions during engagement potentially helping to broaden coping repertoires and consolidate enduring resources over time (Reschly et al., 2008). Taken together, the present findings position proactive engagement as a developmentally constructive mode of academic coping, suggesting that everyday investments in effort and support mobilization may accumulate into broader advantages in students’ motivation, competence development, and well-being.

### 4.3. Defensive Disengagement: Conditional Adaptation and Functional Complexity

Defensive disengagement reflects an effortful, loss-containment coping attempt that emerges when individuals perceive their resources as insufficient. In the MMC-SF, it is indexed by the first-order families of confusion, concealment, self-pity, rumination, and projection. In line with COR principles, defensive disengagement can be understood as an attempt to prevent further loss when depletion is threatened, shifting resources from task engagement toward internal regulation under low controllability. The Adaptive Calibration Model (Del Giudice et al., 2011) similarly frames it as a calibrated response in high-threat, low-control contexts, underscoring that its implications depend on strategy-situation fit, particularly perceived controllability.

Related work suggests that in high-threat, low-control contexts, threat-focused defensive regulation may offer short-term emotional containment; however, when it becomes a default response across situations or is poorly matched to controllability, it can be associated with worse mood and adjustment over time (Wadsworth, 2015). The long-term cost of this regulatory strategy is reflected in our longitudinal data: in sustained academic settings, defensive disengagement was associated with lower later self-efficacy and higher burnout, consistent with persistent resource depletion over time.

### 4.4. Escape Coping: Goal Disengagement and Context-Bound Regulation

In the MMC-SF, escape coping is indexed by its single family and is best conceptualized as goal disengagement specifically via commitment relinquishment (Wrosch et al., 2003), a process that functions to conserve and reallocate psychological resources within the COR framework (Hobfoll, 1989). Its items primarily reflect cognitive disengagement and goal devaluation (e.g., “I tell myself it’s not such a big deal”) rather than behavioral avoidance.

When students perceive continued engagement to be unfeasible or too costly, a regulatory coping response is triggered. This process realigns aspirations with situational constraints through cognitive and emotional adjustment (Brandtstädter & Rothermund, 2002). Specifically, it enables temporary disengagement from unsustainable goals, thereby conserving resources and creating conditions for potential reengagement. This disengagement-reengagement dynamic offers a compelling explanation for the lower temporal stability and ICC observed in the study. These metrics are more likely to reflect the fluid, context-dependent nature of this coping strategy than mere measurement error (Schwartz & Daltroy, 1999).

Recent work indicates that the consequences of escape-like decommitment depend on boundary conditions, including goal attainability and desirability, and individuals’ capacity to reengage in alternative goals (Barlow et al., 2020; Scholer et al., 2024). Disengaging from unattainable goals has been linked to better health and more normative diurnal cortisol patterns (Wrosch et al., 2007). Our findings also suggest that escape coping can be understood as a situationally deployed process with modest and time-limited implications for later adjustment, indicating that temporary disengagement may serve adaptive functions under specific contextual constraints.

Culture shapes an individual’s perception of both resources and threats. Consequently, coping with stress is fundamentally a culturally moderated process: one’s background directly influences the way meaning is constructed, thereby determining which coping strategies are activated and considered effective in a given context (Hobfoll, 2001). For instance, avoidance coping buffered the stress-maladjustment link among Chinese American adolescents, but not European American youth (Jose & Huntsinger, 2005). Similarly, cross-cultural studies on emotion regulation show that, compared to European Americans, emotion suppression yields neutral (Soto et al., 2011) or even positive outcomes (Wei et al., 2013) in harmony-oriented cultures.

Overall, coping with stress is a dynamic resource cycle, driven by continuous appraisal, strategic investment, and contextual feedback of personal and environmental assets. This cycle is embedded within specific situational contexts, where the interaction between the individual and their environment defines critical resources and shapes the strategies that can be effectively use to regulate them. The effectiveness of any coping effort should be determined by its ability to preserve or enhance the individual’s resource base in the immediate ecological context.

### 4.5. Implications

A key theoretical contribution of the MMC-SF is its integration of coping within the framework of Conservation of Resources theory, offering a more nuanced and dynamic understanding of coping in academic contexts. It organizes coping behaviors around context-sensitive resource regulation processes, thereby moving beyond the traditional adaptive–maladaptive binary. Within this framework, proactive engagement primarily supports resource acquisition and investment, defensive disengagement primarily serves resource protection and the prevention of further depletion, and escape coping functions as a short-term form of resource reallocation or withdrawal in contexts of heightened threat or severely constrained resources.

This model sharpens a critical functional distinction between defensive disengagement and escape coping. Specifically, defensive disengagement aims to prevent further loss while remaining engaged with the stressor, whereas escape coping withdraws resources from a threatened goal. This approach not only enriches the conceptualization of academic coping but also provides a more comprehensive framework for understanding how Chinese adolescents navigate stress through resource management processes, ultimately highlighting the importance of a functional perspective on coping.

Another contribution lies in the practical application of the MMC-SF, offering a window into students’ coping behaviors from a functional perspective and enabling schools to provide ecological-level support to enhance academic coping by strengthening psychological resources. This ecological framework operates through three pathways: (1) creating a supportive environment through autonomy-supportive teaching, clear structure, and accessible help to build students’ psychological and academic resources, which in turn fosters proactive engagement and creates a positive resource spiral; (2) identifying students who rely on defensive disengagement and enhancing their access to key resources helps replenish reserves and interrupt negative cycles; (3) guiding students requiring restoration a clear, controllable path back to participation. Therefore, the MMC-SF empowers educators to translate observation into actionable insights, enhancing students’ academic coping abilities by strengthening their underlying resource foundations, rather than focusing solely on strategy instruction.

### 4.6. Limitations and Future Directions

At the theoretical level, escape coping in this study focuses primarily on cognitive and emotional disengagement and does not encompass explicit behavioral avoidance (e.g., absenteeism, procrastination). While this conceptual distinction helps differentiate internal regulation from external behavioral strategies, its limited scope constricts the construct’s explanatory power in academic settings. A promising direction for future work would be to explore the integration of behavioral escape, thereby capturing a more complete range of coping responses to stress.

At the empirical level, several aspects of the MMC-SF’s measurement properties and research methodology warrant further attention. First, the scale does not meet the criterion for strict measurement invariance across school stages; thus, comparisons of observed means across stages should be interpreted cautiously. Second, the low test–retest reliability of the escape coping necessitates cautious interpretation of its longitudinal stability; employing shorter measurement intervals in future studies would better capture its dynamic nature. Third, this study relies on self-reported data, which may carry the risk of common method bias; future research could further validate the criterion-related validity of the scale by integrating multi-method data, such as teacher reports or behavioral observations. Finally, while the factor structure was validated across exploratory and validation samples, all data originated from a single cultural context. The model’s generalizability thus needs to be tested with independent, cross-cultural samples.

Moving forward, research on coping can embrace its inherently dynamic and context-embedded nature. By tracing how individuals’ strategies unfold and adapt in relation to shifting personal and situational resources—using intensive longitudinal designs—we can better understand coping not merely as a set of tactics, but as an ongoing, resource-sensitive process of adaptation.

## 5. Conclusions

Grounded in the Conservation of Resources theory, this study refined the MMC into a concise 34-item tool, the MMC-SF, and established a hybrid higher-order framework comprising proactive engagement, defensive disengagement, and a distinct escape coping dimension. This framework highlights the dynamic, resource-regulatory function of coping, moving beyond the traditional adaptive–maladaptive dichotomy to offer a more context-sensitive and functionally oriented perspective for understanding academic adjustment in Chinese adolescents.

## Figures and Tables

**Table 1:** Model Fit Indices for the MMC and MMC-SF Under ESEM and CFA.

Approach	Scale	χ^2^	df	CFI	TLI	RMSEA	SRMR
ESEM	MMC	3760.57	935	0.959	0.935	0.039	0.015
ESEM	MMC-SF	487.28	265	0.998	0.974	0.029	0.011
CFA	MMC	6331.05	1375	0.928	0.923	0.042	0.043
CFA	MMC-SF	1318.60	505	0.980	0.976	0.028	0.037

Notes: χ^2^ = chi-square; df = degrees of freedom; CFI = comparative fit index; TLI = Tucker–Lewis index; RMSEA = root mean square error of approximation; SRMR = standardized root mean square residual; ESEM = exploratory structural equation modeling; MMC-SF = Multidimensional Measure of Coping-Short Form; CFA = confirmatory factor analysis.

**Table 2:** MMC-SF First-Order Factors: Descriptives and Correlations (Sample 2).

First-Order Factor	1	2	3	4	5	6	7	8	9	10	11
1. ST	1.00										
2. HS	0.52 **	1.00									
3. CS	0.47 **	0.58 **	1.00								
4. SE	0.50 **	0.61 **	0.64 **	1.00							
5. CM	0.52 **	0.63 **	0.63 **	0.76 **	1.00						
6. CF	−0.08 **	−0.20 **	−0.17 **	−0.21 **	−0.19 **	1.00					
7. EP	0.11 **	0.11 **	0.11 **	0.16 **	0.13 **	0.34 **	1.00				
8. CN	−0.02	−0.11 **	−0.12 **	−0.15 **	−0.14 **	0.66 **	0.36 **	1.00			
9. SP	0.00	−0.10 **	−0.07 **	−0.10 **	−0.10 **	0.62 **	0.31 **	0.69 **	1.00		
10. RU	−0.02	−0.14 **	−0.10 **	−0.16 **	−0.13 **	0.62 **	0.22 **	0.65 **	0.74 **	1.00	
11. PJ	−0.11 **	−0.08 **	−0.11 **	−0.14 **	−0.16 **	0.57 **	0.29 **	0.55 **	0.53 **	0.54 **	1.00
M	3.13	2.98	3.13	3.11	3.17	2.21	2.53	2.29	2.30	2.34	1.78
SD	0.68	0.74	0.70	0.68	0.68	0.91	0.82	0.88	0.83	0.93	0.86
Skewness	−0.85	−0.43	−0.66	−0.60	−0.68	0.19	−0.20	0.12	0.06	0.05	0.86
Kurtosis	1.16	−0.08	0.45	0.51	0.65	−0.92	−0.50	−0.81	−0.65	−0.96	−0.18
α	0.89	0.89	0.91	0.90	0.92	0.93	0.82	0.93	0.93	0.94	0.94
ICC	0.39	0.41	0.49	0.43	0.48	0.52	0.25	0.43	0.49	0.58	0.52

Note: Values below the diagonal represent Pearson’s correlations. MMC-SF = Multidimensional Measure of Coping-Short Form; M = mean; SD = standard deviation; α = Cronbach’s alpha. Intraclass correlation coefficient (ICC) values were computed from the 4-week retest subsample in Sample 2. ST = strategizing; HS = help-seeking; CS = comfort-seeking; SE = self-encouragement; CM = commitment; CF = confusion; EP = escape; CN = concealment; SP = self-pity; RU = rumination; PJ = projection. ** *p* < 0.01.

**Table 3:** Measurement Invariance for the MMC-SF Across Gender and School Level.

Model	χ^2^	df	CFI	TLI	RMSEA	SRMR	ΔCFI	ΔTLI
Panel A. Gender								
Configural	1913.83	1010	0.978	0.974	0.030	0.040		
Metric (loadings)	1975.38	1034	0.977	0.974	0.030	0.042	−0.001	0.000
Scalar (intercepts)	2071.06	1058	0.975	0.972	0.031	0.044	−0.002	−0.002
Strict (residuals)	2138.36	1104	0.975	0.973	0.030	0.063	0.000	0.001
Panel B. School stage (junior vs. senior high school)								
Configural	1624.64	880	0.978	0.973	0.029	0.033		
Metric (loadings)	1707.30	902	0.976	0.972	0.030	0.036	−0.002	−0.001
Scalar (intercepts)	1937.08	924	0.970	0.965	0.033	0.036	−0.006	−0.007
Strict (residuals)	3363.69	968	0.929	0.922	0.049	0.172	−0.041	−0.043

Notes: Δ values are computed relative to the immediately preceding (less constrained) model. χ^2^ = chi-square; df = degrees of freedom; CFI = comparative fit index; TLI = Tucker–Lewis index; RMSEA = root mean square error of approximation; SRMR = standardized root mean square residual; MMC-SF = Multidimensional Measure of Coping-Short Form.

**Table 4:** Pattern Loadings for Higher-Order Solutions.

First- Order	Two-Factor Model		Three-Factor Model			Four-Factor Model			
	F1	F2	F1	F2	F3	F1	F2	F3	F4
ST	0.59		0.65			0.50			
HS	0.67		0.65					0.98	
CS	0.67		0.67			0.60			
SE	0.86		0.84			0.86			
CM	0.80		0.80			0.80			
CF		0.66		0.62			0.61		
EP	0.36				0.49	0.32			0.53
CN		0.73		0.67			0.66		
SP		0.85		0.81			0.81		
RU		0.74		0.88			0.88		
PJ		0.60		0.48	0.38		0.48		0.35

Notes: Loadings < 0.30 are omitted (blank cells). Higher-order factors are labeled F1–F4. ST = strategizing; HS = help-seeking; CS = comfort-seeking; SE = self-encouragement; CM = commitment; CF = confusion; EP = escape; CN = concealment; SP = self-pity; RU = rumination; PJ = projection.

**Table 5:** Model Fit Indices for Competing Higher-Order Models.

Model	χ^2^	df	CFI	TLI	RMSEA	SRMR	AIC	BIC
M1	2115.06	548	0.950	0.945	0.046	0.071	87,446.40	88,055.03
M2	1964.85	547	0.955	0.951	0.044	0.053	87,298.19	87,912.02
M3	1963.61	545	0.955	0.950	0.044	0.053	87,300.95	87,925.18

Note: M1 = two-factor solution; M2 = three-factor solution; M3 = four-factor solution. χ^2^ = chi-square; df = degrees of freedom; CFI = comparative fit index; TLI = Tucker–Lewis index; RMSEA = root mean square error of approximation; SRMR = standardized root mean square residual; AIC = Akaike Information Criterion; BIC = Bayesian Information Criterion.

**Table 6:** Hierarchical Regression Models Examining Baseline-Adjusted Prospective Associations Between Higher-Order Coping Dimensions and Academic Self-Efficacy and Burnout.

Predictor	Self-Efficacy (1 Month) β	Self-Efficacy (6 Months) β	Burnout (1 Month) β	Burnout (6 Months) β
Proactive Engagement	0.192 ***	0.155 **	−0.078	−0.091
Defensive Disengagement	−0.182 ***	−0.165 ***	0.230 ***	0.201 ***
Escape coping	0.120 **	0.026	−0.063	−0.076
Baseline outcome	0.317 ***	0.352 ***	0.396 ***	0.292 ***

Notes: All coefficients are standardized regression weights (β). Baseline outcome = T1 self-efficacy for self-efficacy models and T1 burnout for burnout models. 1 Month = T1 to T2 (1-month interval); 6 Months = T1 to T3 (6-month interval). ** *p* < 0.01, *** *p* < 0.001.

## Data Availability

The data presented in this study are available on request from the corresponding author.

## Abbreviations

MMC	The Multidimensional Measure of Coping
MMC-SF	The Multidimensional Measure of Coping-Short Form
ST	Strategizing
HS	Help-seeking
CS	Comfort-seeking
SE	Self-encouragement
CM	Commitment
CF	Confusion
EP	Escape
CN	Concealment
SP	Self-pity
RU	Rumination
PJ	Projection

## Appendix A

Appendix A Table A1 displays the standardized factor loadings for the retained items. Only primary loadings are shown, with the blank cells indicating negligible loading. The items exhibited strong loadings on their intended factors with minimal cross-loadings.

**Table A1:** ESEM Standardized Factor Loadings for MMC-SF Items.

Item	ST	HS	CS	SE	CM	CF	EP	CN	SP	RU	PJ
2	0.619										
3	0.811										
4	0.677										
8		0.793									
9		0.917									
10		0.473									
12			0.691								
13			0.745								
14			0.777								
15			0.635								
18				0.678							
19				0.757	0.315						
20				0.626	0.326						
22				0.344	0.657						
23					0.774						
25					0.690						
28						0.758					
29						0.836					
30						0.716					
32							0.816				
33							0.888				
34							0.730				
37								0.751			
38								0.803			
39								0.749			
41									0.584		
42									0.705		
45									0.569	0.332	
47										0.745	
49										0.767	
50										0.679	
52											0.856
53											0.903
55											0.795

Note: ESEM = exploratory structural equation modeling; MMC-SF = Multidimensional Measure of Coping-Short Form; ST = strategizing; HS = help-seeking; CS = comfort-seeking; SE = self-encouragement; CM = commitment; CF = confusion; EP = escape; CN = concealment; SP = self-pity; RU = rumination; PJ = projection.
